# Cutaneous Dermal Metastasis of Inflammatory Breast Carcinoma Mimicking Granuloma Annulare

**DOI:** 10.7759/cureus.47544

**Published:** 2023-10-23

**Authors:** Vikram Shaw, Jay Patel, Hanna Siatecka, Omid Jalali

**Affiliations:** 1 College of Medicine, Baylor College of Medicine, Houston, USA; 2 Department of Dermatology, Baylor College of Medicine, Houston, USA; 3 Department of Pathology and Immunology, Baylor College of Medicine, Houston, USA

**Keywords:** targetoid lesion, inflammatory breast carcinoma, breast cancer, granuloma annulare, cutaneous dermal metastasis

## Abstract

Breast cancer can present as a wide range of cutaneous lesions at the time of diagnosis or months to years after a known diagnosis of breast cancer. Cutaneous sequela of breast cancer, including metastasis, have a diverse range of clinical appearances. Here, we describe the case of a 59-year-old female with stage IV metastatic inflammatory breast carcinoma presenting with a chronic worsening rash on her anterior chest wall. Biopsy results demonstrated metastatic carcinoma cells within the dermal lymphatics, consistent with primary breast cancer. To our knowledge, based on a thorough review of the literature, no previous case reports detailing cutaneous metastasis of breast cancer have identified a rash mimicking granuloma annulare. The present case highlights the importance of early dermatologic referral if any abnormal or persistent lesions appear in a patient with a history of or current treatment for breast cancer.

## Introduction

Breast cancer can initially present as cutaneous lesions or subsequently after a known diagnosis of breast cancer months to years later. Cutaneous sequela, including metastasis, of breast cancer can manifest in a large number of ways, including urticarial, targetoid, and melanotic lesions [1]. Herein, we report a case of a cutaneous breast cancer metastasis presenting as a rash mimicking granuloma annulare.

## Case presentation

**Figure 1:**
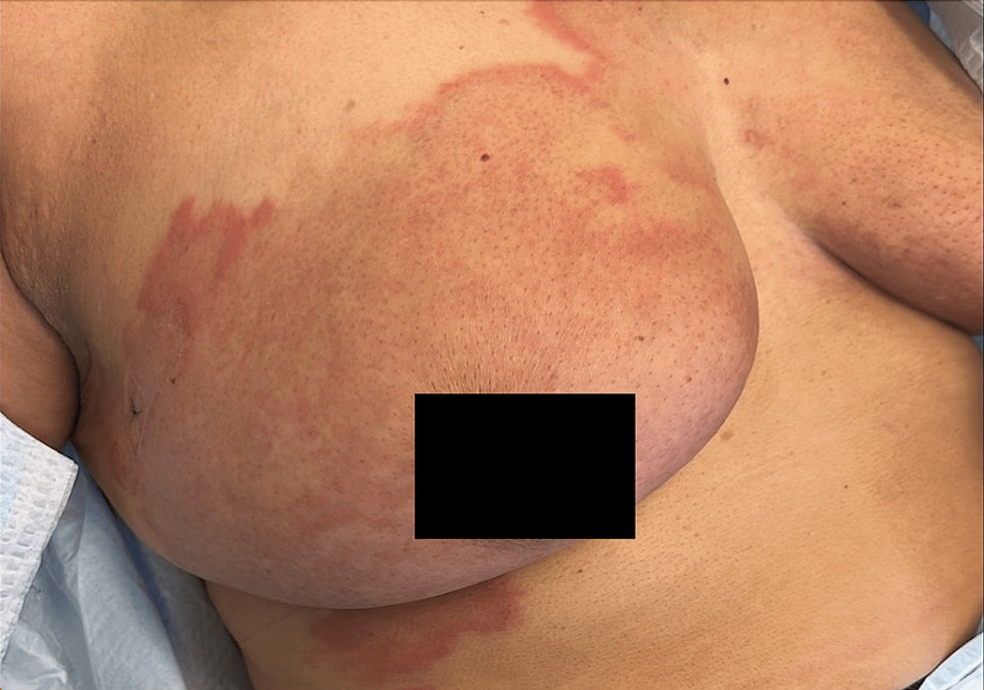
Confluent erythematous annular plaques with faint pink center on anterior chest and right breast found to be cutaneous metastasis of inflammatory breast carcinoma.

A 59-year-old female with stage IV metastatic inflammatory breast carcinoma presented to the dermatology clinic for a subacute on chronic worsening of a rash on her anterior chest wall (Figure 1). 

Seven years prior, she was diagnosed with ductal carcinoma in situ (DCIS) but was lost to follow-up for one year. At the subsequent visit, she was noted to have progression to lymph node-positive, ER-negative, PR-negative, and HER2-positive invasive ductal carcinoma (IDC). The patient received six rounds of docetaxel, trastuzumab, and pertuzumab (THP) and was then transitioned to the AVIATOR trial (NCT03414658) and received trastuzumab, vinorelbine, and avelumab. At this point, three years after the initial diagnosis, oligometastatic disease was noted in the right axilla. Additionally, avelumab was stopped due to newly diagnosed scleroderma. The patient received radiation therapy to the metastatic site and was taken off the AVIATOR trial for progressive axillary node involvement noted one year prior to the current presentation. The patient continued on a regimen of trastuzumab and vinorelbine. An MRI brain two months prior to presentation was without intracranial metastases and a nuclear medicine bone scan one month prior to presentation showed no evidence of metastatic disease. The patient was referred to dermatology after mentioning that the rash on her breasts was getting worse and were now very pruritic.

The patient reported she had a small rash on both of her breasts present for the past two years that waxed and waned in appearance. Over the past six months, however, the rash became more pruritic and increased in surface area over her breasts and now “jumped” to her upper abdomen and under her right breast, making her worried she was using something topical that was resulting in the rash. On physical exam, there were multiple, confluent erythematous plaques in an annular configuration on her anterior chest, right breast, and right inframammary area with light pink coloration in the center and a few smaller areas on her left breast, mimicking the pattern of granuloma annulare.

**Figure 2:**
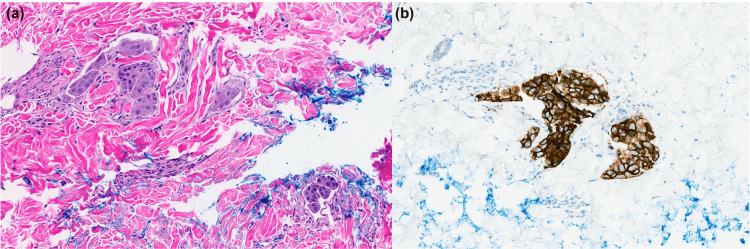
Histopathology of cutaneous metastasis of inflammatory breast carcinoma. (a) Breast carcinoma histopathology via an H&E stain at 400x magnification. (b) HER2+ stained cells within the dermal lymphatics at 400x magnification.

A skin biopsy was recommended for further evaluation and the patient agreed to proceed. A 4 mm punch biopsy was performed, and histopathological evaluation demonstrated metastatic carcinoma cells within the dermal lymphatics, consistent with primary breast cancer (Figure 2). This represents the progression of her cancer despite unchanged findings on imaging and she will be switched to a different chemotherapy regimen at her next oncology visit.

## Discussion

Cutaneous metastases of breast cancer can present with a diverse range of cutaneous lesions, making early clinical recognition important. A search of the literature and associated clinical images revealed that the vast majority of annular lesions described were erythematous patches, targetoid lesions, or appeared like erythema annulare centrifugum, with none mentioning lesions mimicking the granuloma annulare seen in our case with the exam demonstrating a characteristic rash and an absence of visible scale [1-4]. This case highlights the importance of consideration of cutaneous metastasis of breast cancer if one sees lesions that appear like granuloma annulare in a patient with a history of breast cancer. In addition, this emphasizes the importance of early dermatologic referral if any abnormal or persistent lesions appear while a patient is being treated for breast cancer as it has a large number of cutaneous manifestations and is a marker for the progression of the disease, even if there is negative radiographic evidence of metastatic disease [3].

## Conclusions

A high index of suspicion for cutaneous metastasis should exist in breast cancer patients with abnormal or persistent lesions, and early dermatologic referral can have important implications in the diagnosis of metastasis, which can be followed up promptly with oncologic management. By virtue of this case report, we hope to encourage others to report imaging and histology findings for cutaneous metastasis of breast cancer to develop a clearer picture of the full, diverse range of cutaneous manifestations. By understanding, cataloging, and publishing the range of cutaneous manifestations, we hope to improve the ability of clinicians to recognize and diagnose these lesions with the ultimate goal of improving patient care. 
